# Data for the synthesis and characterisation of 2,6-di(bromomethyl)-3,5-bis(alkoxycarbonyl)-4-aryl-1,4-dihydropyridines as important intermediates for synthesis of amphiphilic 1,4-dihydropyridines

**DOI:** 10.1016/j.dib.2020.105532

**Published:** 2020-04-12

**Authors:** Martins Rucins, Klavs Pajuste, Arkadij Sobolev, Mara Plotniece, Nadiia Pikun, Karlis Pajuste, Aiva Plotniece

**Affiliations:** a Department of Membrane Active Compounds, Latvian Institute of Organic Synthesis, Aizkraukles str. 21, LV-1006, Riga, Latvia; b Department of Pharmaceutical Chemistry, Faculty of Pharmacy, Riga Stradiņš University, Dzirciema str. 16, LV-1007, Riga, Latvia

**Keywords:** Synthesis, Azines, 1,4-dihydropyridines, 2,6-di(bromomethyl)-1,4-dihydropyridines, Bromination, NMR data

## Abstract

This data file describes the synthetic protocol for preparation of the original 2,6-di(bromomethyl)-3,5-bis(alkoxycarbonyl)-4-aryl-1,4-dihydropyridines. In total, 6 unpublished compounds were obtained and characterised. The 2,6-di(bromomethyl)-1,4-dihydropyridines are mainly used as intermediates for synthesis of various lipid-like compounds based on 1,4-dihydropyridine cycle. All the structures of 2,6-di(bromomethyl)-1,4-dihydropyridines were confirmed by Nuclear Magnetic Resonance (NMR, including ^1^H NMR and ^13^C NMR) data. The data provided herein are directly related to the previously published research article – “Novel cationic amphiphilic 1,4-dihydropyridine derivatives for DNA delivery” [1] where three derivatives (2,6-di(bromomethyl)-4-phenyl-1,4-dihydropyridines **2a-c**) from six presented in this data file were used as starting materials in synthesis of amphiphilic 1,4-dihydropyridines without any purification and characterisation. Synthesis of other three 2,6-di(bromomethyl)-3,5-bis(alkoxycarbonyl)-4-aryl-1,4-dihydropyridines **2d-f** and their characterisation are reported herein at the first time. Information provided in this data file can be used in organic synthesis by other chemists to develop synthetic strategies for the construction of various cationic 1,4-dihydropyridine derivatives and related heterocycles.

Specifications table

**Table:** 

Subject	Chemistry
Specific subject area	Organic chemistry, bromination
Type of data	Synthetic scheme, general protocol for synthesis, table with structures, NMR data; in supplementary data – NMR spectra
How data were acquired	^1^H NMR spectra were recorded with a Bruker Fourier (300 MHz) or a Bruker Avance Neo (400 MHz) spectrometer and ^13^C NMR spectra were recorded with a Bruker Avance Neo (100 MHz) spectrometer. The coupling constants are expressed in Hertz (Hz). The chemical shifts of the hydrogen and carbon atoms are presented in parts per million (ppm) and referred to the residual signals of the non-deuterated CDCl_3_ (δ: 7.26) solvent for ^1^H NMR spectra and CDCl_3_ (δ: 77.0) solvent for ^13^C NMR, respectively. Multiplicities are abbreviated as s: singlet; t: triplet; m: multiplet; br: broad; td: triplet of doublets.
Data format	Raw and analysed
Parameters for data collection	Data was collected for characterisation purposes. After purification of 2,6-di(bromomethyl)−1,4-dihydropyridines with flash-chromatography, ^1^H and ^13^C NMR data was collected.
Description of data collection	Data was collected via the raw output files from the respective hardware. ^1^H and ^13^C NMR spectra were recorded as fid files.
Data source location	Latvian Institute of Organic Synthesis, Riga, Latvia
Data accessibility	Data is provided within the article.
Related research article	Hyvönen Z.; Plotniece A.; Reine I.; Chekavichus B.; Duburs G.; Urtti A. Novel cationic amphiphilic 1,4–dihydropyridine derivatives for DNA delivery. *Biochim. Biophys. Acta*, 2000, 1509, 451–466. doi:10.1016/S0005–2736(00)00,327–8

## Value of the data

• The data contains the general synthetic procedure for bromination of the methyl groups of 3,5-bis(alkoxycarbonyl)−2,6-dimethyl-4-aryl-1,4-dihydropyridine which may serve as valuable guidance for organic chemists.
• The obtained 2,6-di(bromomethyl)−1,4-dihydropyridines can be used as intermediates for synthesis of various lipid-like compounds based on 1,4-dihydropyridine cycle.
• The data provides characterisation of original compounds - 2,6-di(bromomethyl)−3,5-bis(alkoxycarbonyl)−4-aryl-1,4-dihydropyridines which have not been reported before.
• Additionally, described synthetic procedure and obtained spectral data will be useful for preparation and structure elucidation of brominated derivatives in related heterocyclic systems.
• Besides the use of 2,6-di(bromomethyl) derivatives in synthesis of cationic 1,4-dihydropyridines their applications may be extended to other reactions of bromomethyl groups.

## 1 Data description

**Fig. 1:**
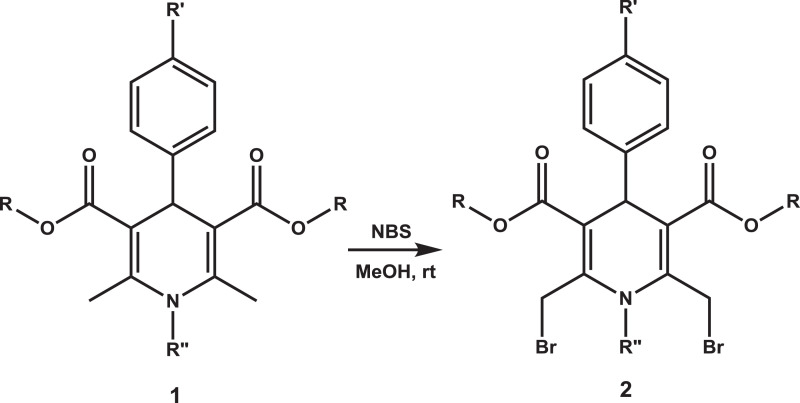
Scheme of the synthesis of 2,6-di(bromomethyl)−1,4-dihydropyridines **2** from the corresponding 3,5-bis(alkoxycarbonyl)−2,6-dimethyl-4-phenyl-1,4-dihydropyridines **1** in the reaction with N-bromosuccinimide (NBS). Structures of new 2,6-di(bromomethyl)−1,4-dihydropyridines **2a-f** (Table 1).

Bromination of the methyl groups of 2,6-dimethyl-1,4-dihydropyridines is a key step in procedure of synthesis of 1,4-dihydropyridines with cationic moieties for further biological studies [1,2]. The dataset presents the synthesis and physicochemical characterisation of 2,6-di(bromomethyl)−3,5-bis(alkoxycarbonyl)−4-aryl-1,4-dihydropyridines **2** which data have not been described before. Previously 2,6-di(bromomethyl)−3,5-bis(alkoxycarbonyl)−4-phenyl-1,4-dihydropyridines **2a-c** were applied for synthesis of amphiphilic 1,4-dihydropyridines (1,4-DHP) without any purification and identification [1]. Synthesis of other 2,6-di(bromomethyl)−3,5-bis(alkoxycarbonyl)−4-aryl-1,4-dihydropyridines **2d-f** is reported herein. These compounds **2a-f** are prepared for further use in synthesis of cationic 1,4-DHPs and other modifications giving new sources of biologically active compounds. Parent 2,6-dimethyl-1,4-DHPs **1a-c** [1] and **1d-f** [3] were synthesised by the previously described procedures. The synthetic scheme and also the structures of all 2,6-di(bromomethyl)−1,4-dihydropyridines are depicted in Fig. 1. Structures of new 2,6-di(bromomethyl)−1,4-dihydropyridines **2** (Table 1) were confirmed by ^1^H and ^13^C NMR spectra.

**Table 1:** Structures of compounds.

Nr	R	R’	R”
**a**	C_14_H_29_-n	H	H
**b**	C_16_H_33_-n	H	H
**c**	C_12_H_25_-n	H	CH_3_
**d**	C_14_H_29_-n	OH	H
**e**	C_10_H_24_-n	OCH_3_	H
**f**	C_10_H_24_-n	OC_7_H_15_-n	H

## 2 Experimental design, materials and methods

### 2.1 General information

All reagents and solvents were purchased from commercial suppliers (ACROS, Sigma-Aldrich) and used without further purification. Organic solutions were concentrated under reduced pressure on a rotary evaporator (Buchi). Reactions were monitored with thin-layer chromatography (TLC) on Silica gel 60 F254 aluminium sheets 20 × 20 cm; eluent: EtOAc:hexane=1:5. Flash column chromatography was accomplished using flow chromatography (Armen Instrument). Silica gel (Merck, Silica gel 60, 35–70 µm) was used for column chromatography with gradient elution: EtOAc:hexane 0:100 to 15:85%. Elemental analyses were determined on an Elemental Combustion System ECS 4010 (Costech Instruments). Melting points were determined on an OptiMelt (SRS Stanford Research Systems).

### 2.2 General procedure for synthesis of 2,6-di(bromomethyl)−1,4-dihydropyridines 2

To a solution of 2,6-dimethyl-1,4-DHP **1a-f** (5.00 mmol) in methanol (100 mL) a solution of NBS (10.40 mmol) in methanol (50 mL) was added dropwise. The reaction mixture was stirred at rt for 2 h, completion of the reaction was monitored by TLC.

In the case of comp. **2a,b** the yellow precipitate was filtered off and washed with water; for the comp. **2c-f** the solvent was evaporated and the residue was purified by flash-chromatography. The compounds **2d-f** compounds were found to be unstable during the storage due to lactonisation into 8-aryl-5,8-dihydro-1*H*,3*H*-difuro[3,4-*b*:3′,4′-*e*]pyridine-1,7(4*H*)‑dione derivatives [4]. Satisfactory NMR data were obtained after two purifications by chromatography.

*Ditetradecyl 2,6-bis(bromomethyl)−4-phenyl-1,4-dihydropyridine-3,5-dicarboxylate* (**2a**)



Yield: 73%. Yellow powder; m.p. 84–86 °C; ^1^H NMR (300 MHz, CDCl_3_) δ: 7.28 – 7.14 (m, 5H) overlap with CHCl_3_, 6.45 (s, 1H), 5.02 (s, 1H), 4.93 and 4.63 (AB-system, *J* = 11.6 Hz, 4H), 4.12 (td, *J* = 6.6, 2.7 Hz, 4H), 1.66 – 1.56 (m, 4H), 1.26 (s, 44H), 0.92 – 0.85 (m, 6H) ppm. ^13^C NMR (100 MHz, CDCl_3_) δ: 166.40, 145.78, 141.78, 128.40, 128.10, 127.03, 106.25, 64.95, 40.26, 32.08, 29.87, 29.85, 29.83, 29.79, 29.72, 29.53, 29.44, 28.73, 27.52, 26.21, 22.85, 14.28 ppm. Anal. Calcd. for C_43_H_69_Br_2_NO_4_: C%, 62.69; H%, 8.44; N%, 1.70. Found: C%, 62.67; H% 8.45; N% 1.67.

*Dihexadecyl 2,6-bis(bromomethyl)−4-phenyl-1,4-dihydropyridine-3,5-dicarboxylate* (**2b**)



Yield: 53%. Yellow powder; m.p. 86–88 °C; ^1^H NMR (300 MHz, CDCl_3_) δ: 7.28 – 7.15 (m, 5H) overlap with CHCl_3_, 6.44 (s, 1H), 5.02 (s, 1H), 4.93 and 4.63 (AB-system, *J* = 11.6 Hz, 4H), 4.07 (td, *J* = 6.6, 2.7 Hz, 4H), 1.66 – 1.56 (m, 4H), 1.34 – 1.19 (m, 52H), 0.92 – 0.84 (m, 6H) pmm. ^13^C NMR (100 MHz, CDCl_3_) δ: 166.40, 145.78, 141.76, 128.40, 128.10, 127.03, 106.26, 64.95, 40.26, 32.09, 29.88, 29.83, 29.79, 29.73, 29.53, 29.45, 28.73, 27.52, 26.21, 22.85, 14.28 ppm. Anal. Calcd. for C_47_H_77_Br_2_NO_4_: C%, 64.15; H%, 8.82; N%, 1.59. Found: C%, 64.40; H% 8.86; N% 1.57.

*Didodecyl 2,6-bis(bromomethyl)−1-methyl-4-phenyl-1,4-dihydropyridine-3,5-dicarboxylate* (**2c**)



Yield: 48%. Yellow oil; ^1^H NMR (300 MHz, CDCl_3_) δ: 7.23 – 7.13 (m, 5H), 5.24 (s, 1H), 5.03 – 4.92 (m, 2H), 4.88 – 4.77 (m, 2H), 4.16 (t, *J* = 6.6 Hz, 4H), 3.47 (s, 3H), 1.72 – 1.61 (m, 4H), 1.39 – 1.20 (m, 36H), 0.92 – 0.85 (m, 6H) ppm. ^13^C NMR (100 MHz, CDCl_3_) δ: 166.41, 147.39, 144.01, 128.49, 127.00, 126.84, 108.91, 65.16, 37.98, 32.10, 32.07, 29.83, 29.79, 29.76, 29.72, 29.51, 29.43, 28.75, 26.22, 24.95, 22.84, 14.27 ppm.

*Ditetradecyl 2,6-bis(bromomethyl)−4-(4-hydroxyphenyl)−1,4-dihydropyridine-3,5-dicarboxylate* (**2d**)



Yield: 17%. Yellow oil; ^1^H NMR (300 MHz, CDCl_3_) δ: 7.14 – 7.09 (m, 2H), 6.70 – 6.65 (m, 2H), 6.42 (br.s, 1H), 4.95 (s, 1H), 4.89 and 4.63 (AB-system, *J* = 11.6 Hz, 4H), 4.58 (br.s, 1H), 4.11 – 4.04 (m, 4H), 1.66 – 1.56 (m, 4H), 1.34 – 1.20 (m, 44H), 0.91 – 0.85 (m, 6H) ppm. ^13^C NMR (100 MHz, CDCl_3_) δ: 166.73, 154.82, 141.73, 138.14, 129.27, 115.26, 106.30, 65.05, 39.35, 32.05, 29.84, 29.82, 29.80, 29.78, 29.71, 29.50, 29.43, 28.69, 27.39, 26.20, 22.82, 14.25 ppm.

*Didecyl 2,6-bis(bromomethyl)−4-(4-methoxyphenyl)−1,4-dihydropyridine-3,5-dicarboxylate* (**2e**)



Yield: 52%. Yellow oil; ^1^H NMR (300 MHz, CDCl_3_) δ: 7.20 – 7.14 (m, 2H), 6.78 – 6.73 (m, 2H), 6.43 (br.s, 1H), 4.96 (s, 1H), 4.90 and 4.64 (AB-system, *J* = 11.5 Hz, 4H), 4.12 – 4.03 (m, 4H), 3.75 (s, 3H), 1.67 – 1.56 (m, 4H), 1.34 – 1.22 (m, 28H), 0.91 – 0.85 (m, 6H) ppm. ^13^C NMR (100 MHz, CDCl_3_) δ: 166.49, 158.61, 141.59, 138.30, 129.13, 113.71, 106.36, 64.91, 55.27, 39.34, 32.98, 32.04, 29.72, 29.47, 29.44, 28.73, 27.50, 26.21, 22.83, 14.25 ppm.

*Didecyl 2,6-bis(bromomethyl)−4-(4-(heptyloxy)phenyl)−1,4-dihydropyridine-3,5-dicarboxylate* (**2f**)



Yield: 15%. Yellow oil; ^1^H NMR (300 MHz, CDCl_3_) δ: 7.17 – 7.12 (m, 2H), 6.77 – 6.72 (m, 2H), 6.41 (br.s, 1H), 4.95 (s, 1H), 4.91 and 4.63 (AB-system, *J* = 11.5 Hz, 4H), 4.12 – 4.02 (m, 4H), 3.88 (t, *J* = 6.6 Hz, 2H), 1.80 – 1.68 (m, 2H), 1.67 – 1.56 (m, 4H), 1.46 – 1.18 (m, 36H), 0.91 – 0.85 (m, 9H) ppm. ^13^C NMR (100 MHz, CDCl_3_) δ: 166.49, 158.25, 141.41, 138.03, 129.11, 114.27, 106.50, 68.02, 64.90, 39.39, 32.07, 31.94, 29.75, 29.74, 29.50, 29.48, 29.45, 29.23, 28.75, 27.61, 26.23, 26.19, 22.85, 14.27 ppm.

## Declaration of Competing Interest

The author declare that they have no known competing financial interests or personal relationships that could appeared to influence the work reported in this paper.

## Appendix Supplementary materials

Image, application 1

Image, application 2
