# Supplementary material for: Data for the synthesis and characterisation of 2,6-di(bromomethyl)-3,5-bis(alkoxycarbonyl)-4-aryl-1,4-dihydropyridines as important intermediates for synthesis of amphiphilic 1,4-dihydropyridines
Source: Data Brief. 2020 Apr 12;30:105532. doi: 10.1016/j.dib.2020.105532 (PMC7163309; doi:10.1016/j.dib.2020.105532)
Supplement: Supplementary file 1 [file mmc1.pdf]

Data for the synthesis and characterisation of 2,6-di(bromomethyl)-3,5-bis(alkoxycarbonyl)-4-aryl-1,4-dihydropyridines as important intermediates for synthesis of amphiphilic 1,4-dihydropyridines

Authors

Martins Rucins[1], Klavs Pajuste[1], Arkadij Sobolev[1], Mara Plotniece[2], Nadiia Pikun[1], Karlis Pajuste[1], Aiva Plotniece[1,2]

Affiliations

[1] Department of Membrane Active Compounds, Latvian Institute of Organic Synthesis, Aizkraukles str. 21, LV-1006, Riga, Latvia
[2] Department of Pharmaceutical Chemistry, Faculty of Pharmacy, Riga Stradiņš University, Dzirciema str. 16, LV-1007, Riga, Latvia

Corresponding author(s)

Martins Rucins (rucins@osi.lv)

Table of Contents

^{1}H and ^{13}C NMR spectra S2-S13

2a

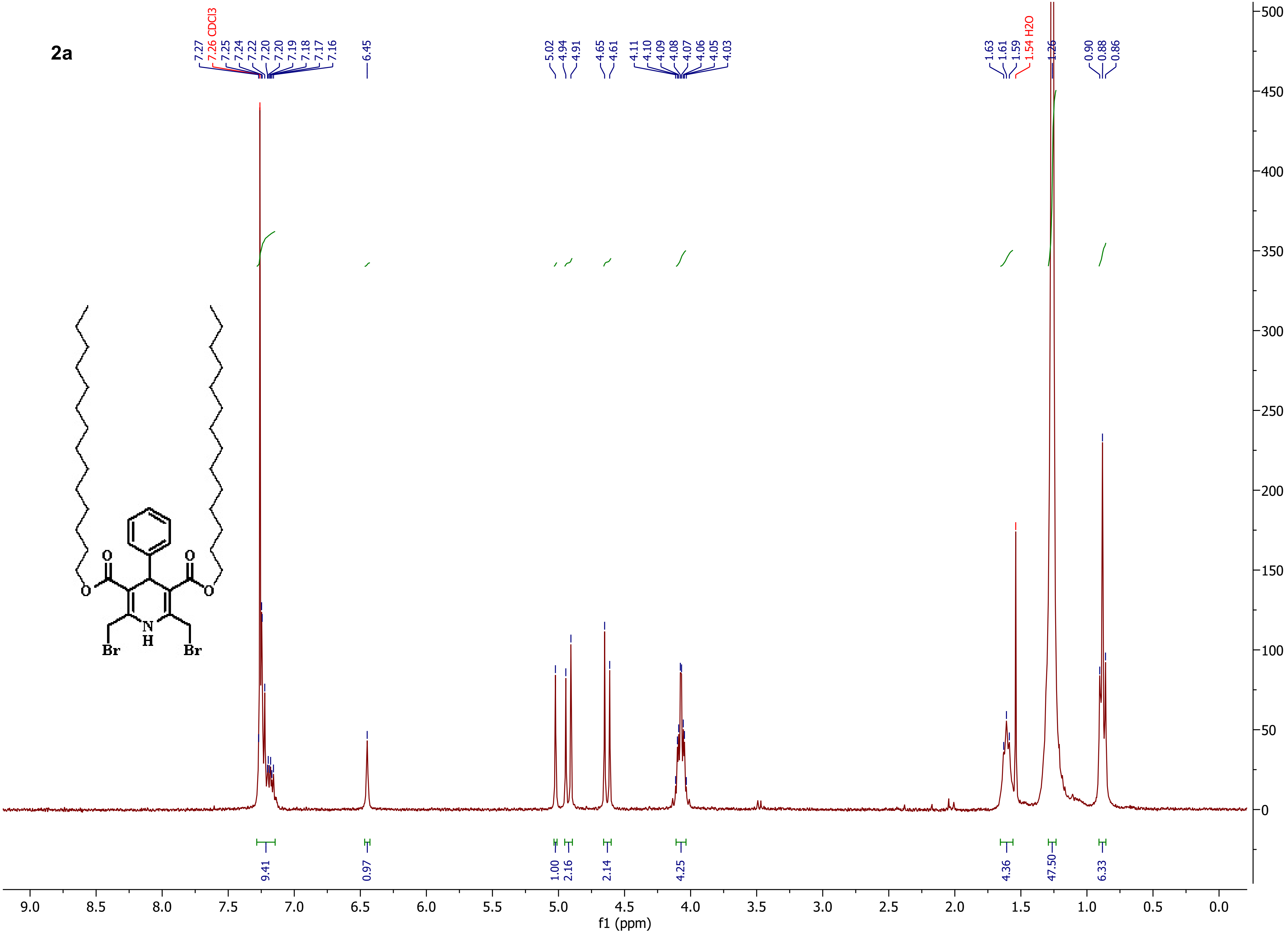
7.27
7.26 CDCl3
7.25
7.24
7.22
7.20
7.20
7.19
7.18
7.17
7.16
6.45
5.02
4.94
4.91
4.65
4.61
4.11
4.10
4.09
4.08
4.07
4.06
4.05
4.03
1.63
1.61
1.59
1.54 H2O
1.26
0.90
0.88
0.86
9.41
0.97
1.00
2.16
2.14
4.25
4.36
47.50
6.33
f1 (ppm)
O
O
N
H
Br
Br

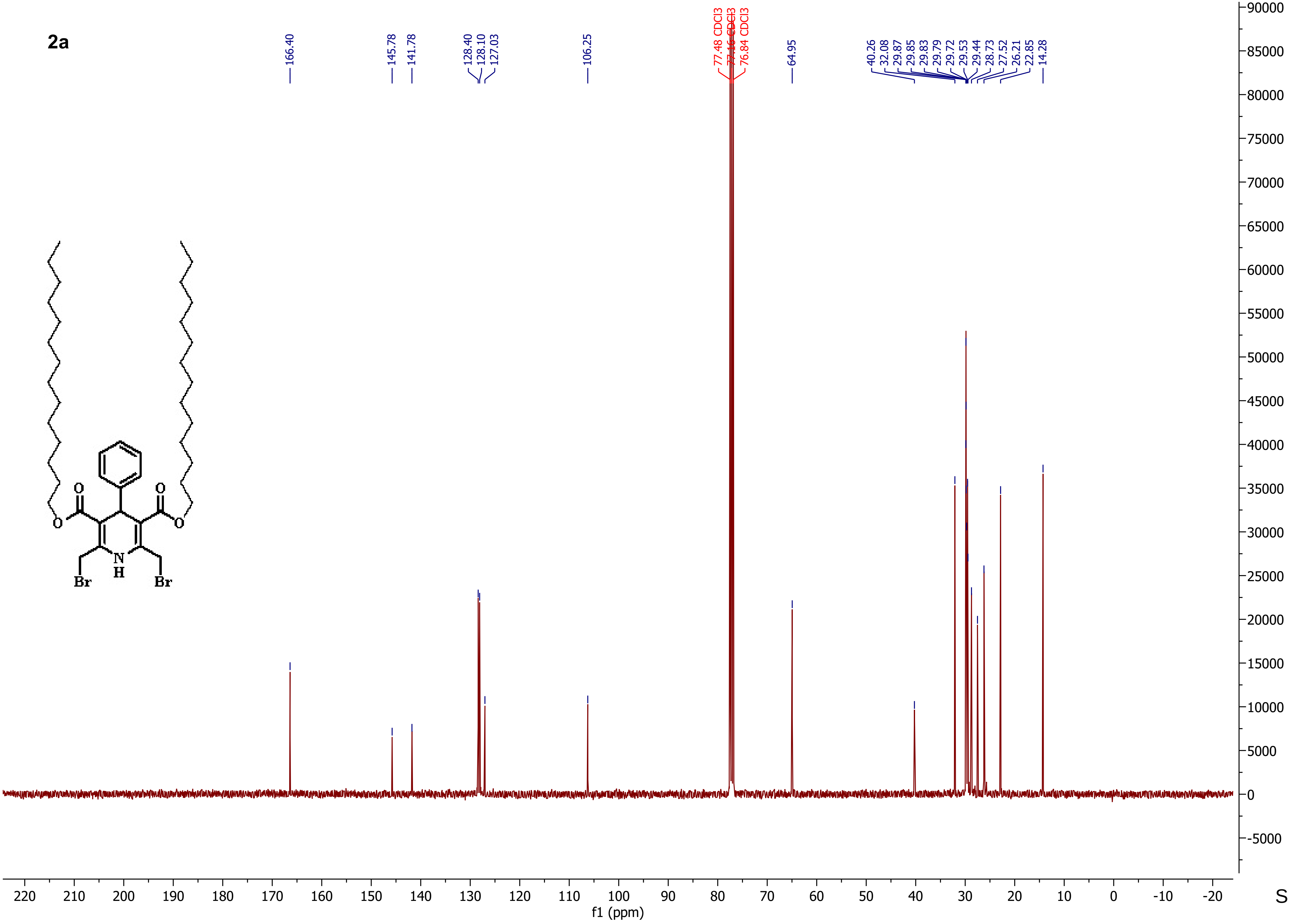

2a
166.40
145.78
141.78
128.40
128.10
127.03
106.25
77.48 CDCl3
77.16 CDCl3
76.84 CDCl3
64.95
40.26
32.08
29.87
29.85
29.83
29.79
29.72
29.53
29.44
28.73
27.52
26.21
22.85
14.28
O
O
O
O
N
H
Br
Br
90000
85000
80000
75000
70000
65000
60000
55000
50000
45000
40000
35000
30000
25000
20000
15000
10000
5000
0
-5000
220 210 200 190 180 170 160 150 140 130 120 110 100 90 80 70 60 50 40 30 20 10 0 -10 -20
f1 (ppm)

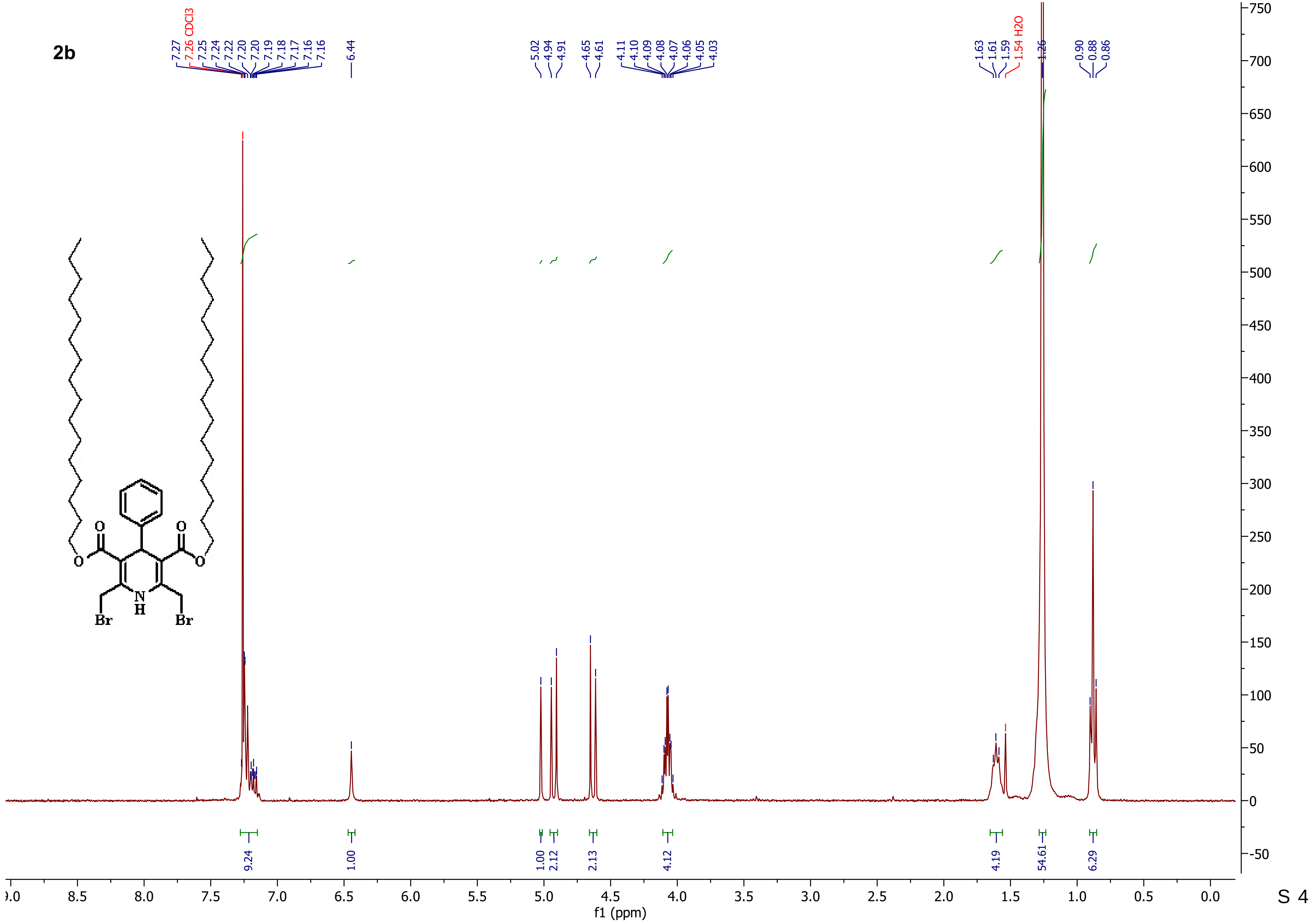
2b
7.27
7.26 CDCl3
7.25
7.24
7.22
7.20
7.20
7.19
7.18
7.17
7.16
7.16
6.44
5.02
4.94
4.91
4.65
4.61
4.11
4.10
4.09
4.08
4.07
4.06
4.05
4.03
1.63
1.61
1.59
1.54 H2O
1.26
0.90
0.88
0.86
9.24
1.00
1.00
2.12
2.13
4.12
4.19
54.61
6.29
f1 (ppm)
O
O
O
O
N
H
Br
Br

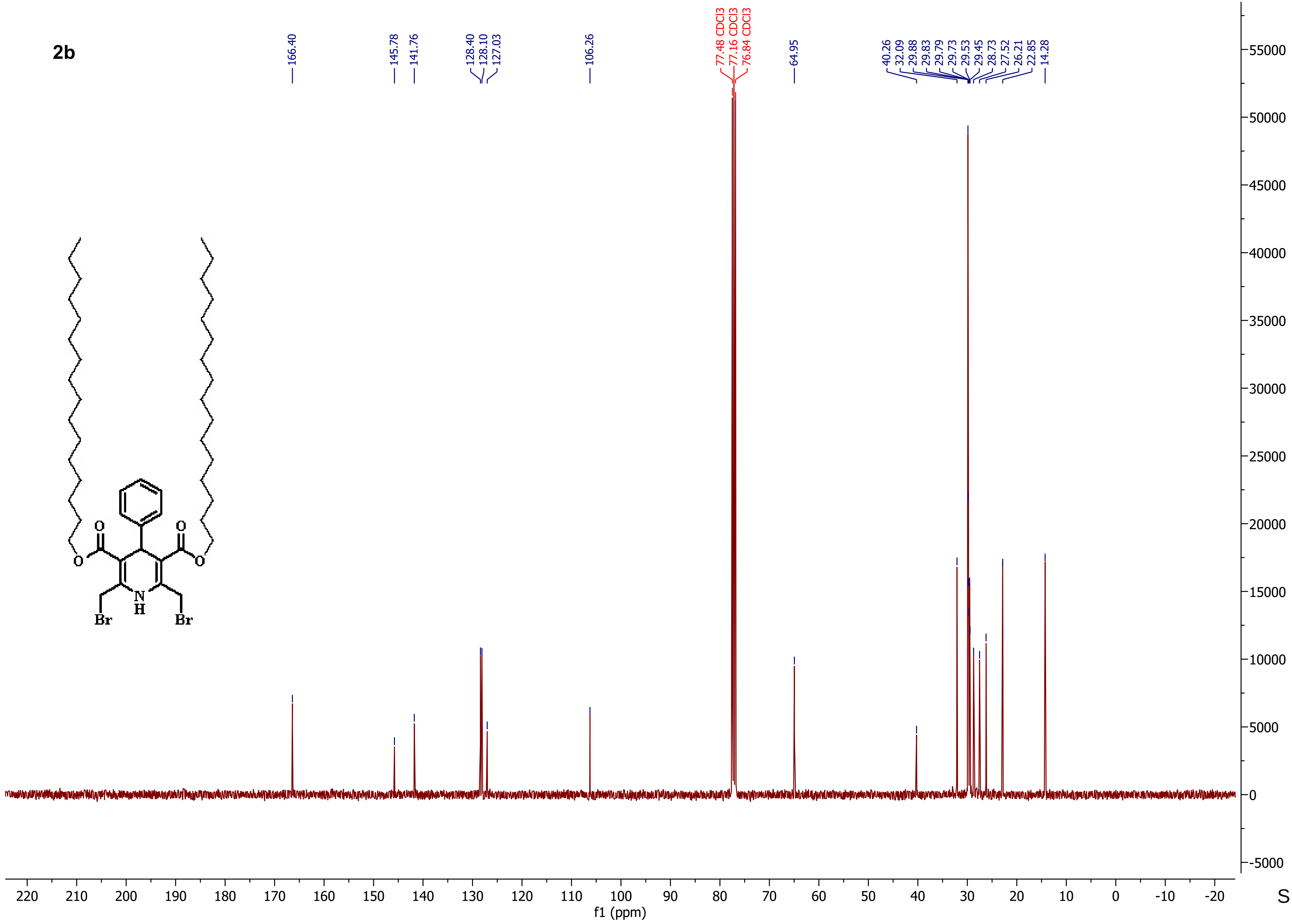
2b
166.40
145.78
141.76
128.40
128.10
127.03
106.26
77.48 CDCl3
77.16 CDCl3
76.84 CDCl3
64.95
40.26
32.09
29.88
29.83
29.79
29.73
29.53
29.45
28.73
27.52
26.21
22.85
14.28
f1 (ppm)
O
O
O
O
N
H
Br
Br

2c

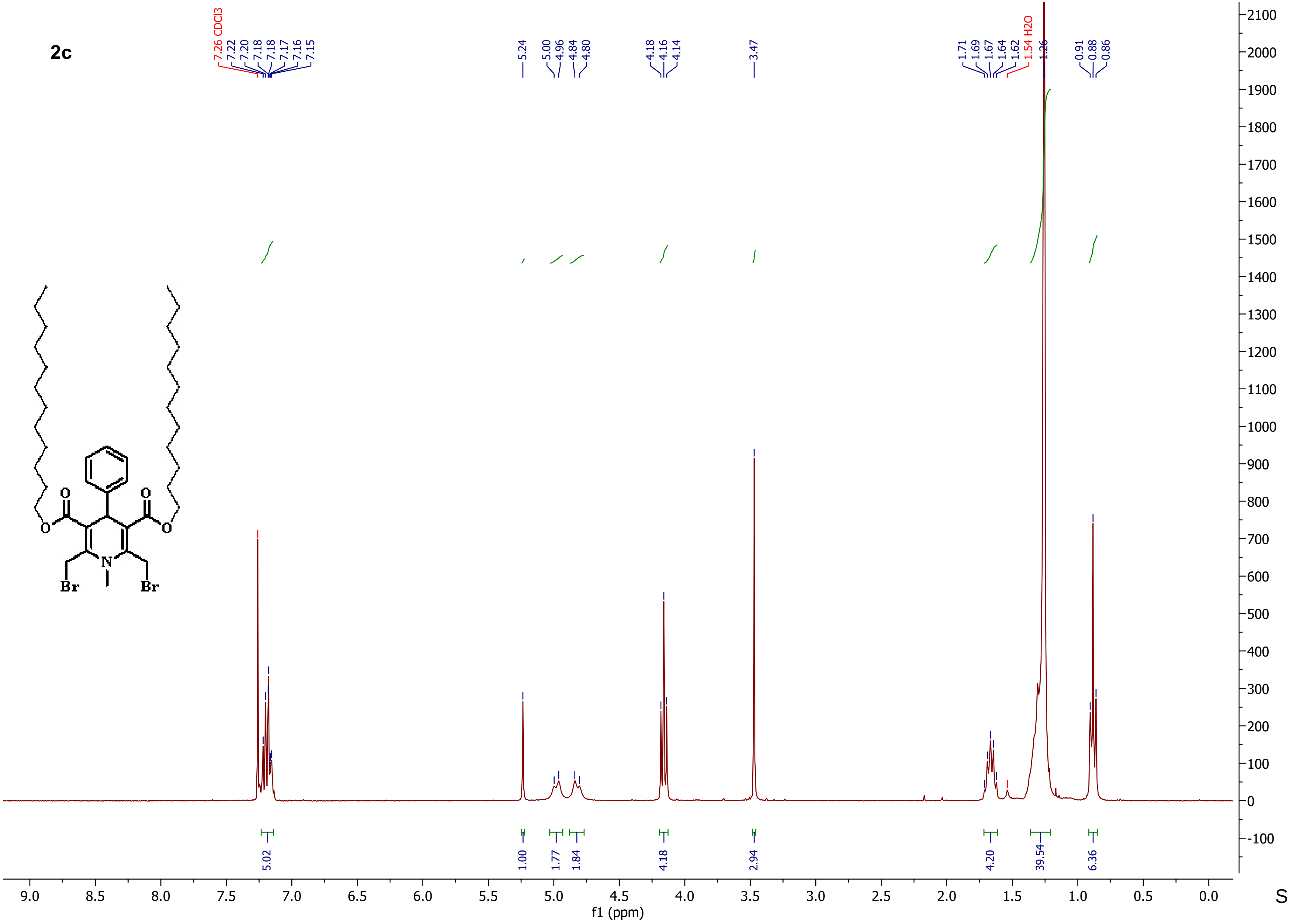
7.26 CDCl3
7.22
7.20
7.18
7.18
7.17
7.16
7.15
5.24
5.00
4.96
4.84
4.80
4.18
4.16
4.14
3.47
1.71
1.69
1.67
1.64
1.62
1.54 H2O
1.26
0.91
0.88
0.86
O
O
O
O
N
Br
Br
5.02
1.00
1.77
1.84
4.18
2.94
4.20
39.54
6.36
f1 (ppm)

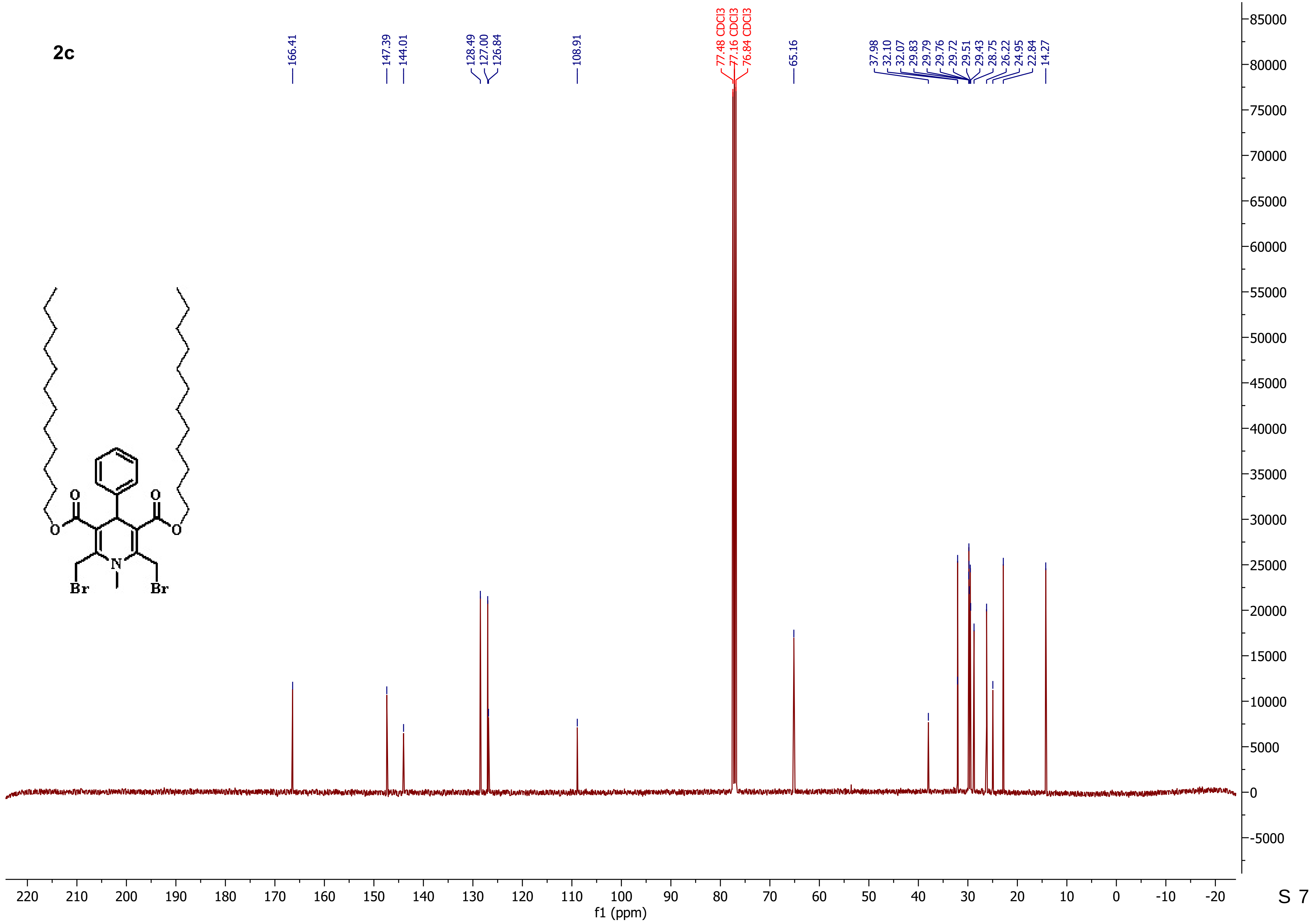
2c
166.41
147.39
144.01
128.49
127.00
126.84
108.91
77.48 CDCl3
77.16 CDCl3
76.84 CDCl3
65.16
37.98
32.10
32.07
29.83
29.79
29.76
29.72
29.51
29.43
28.75
26.22
24.95
22.84
14.27
f1 (ppm)
O
O
O
O
N
Br
Br

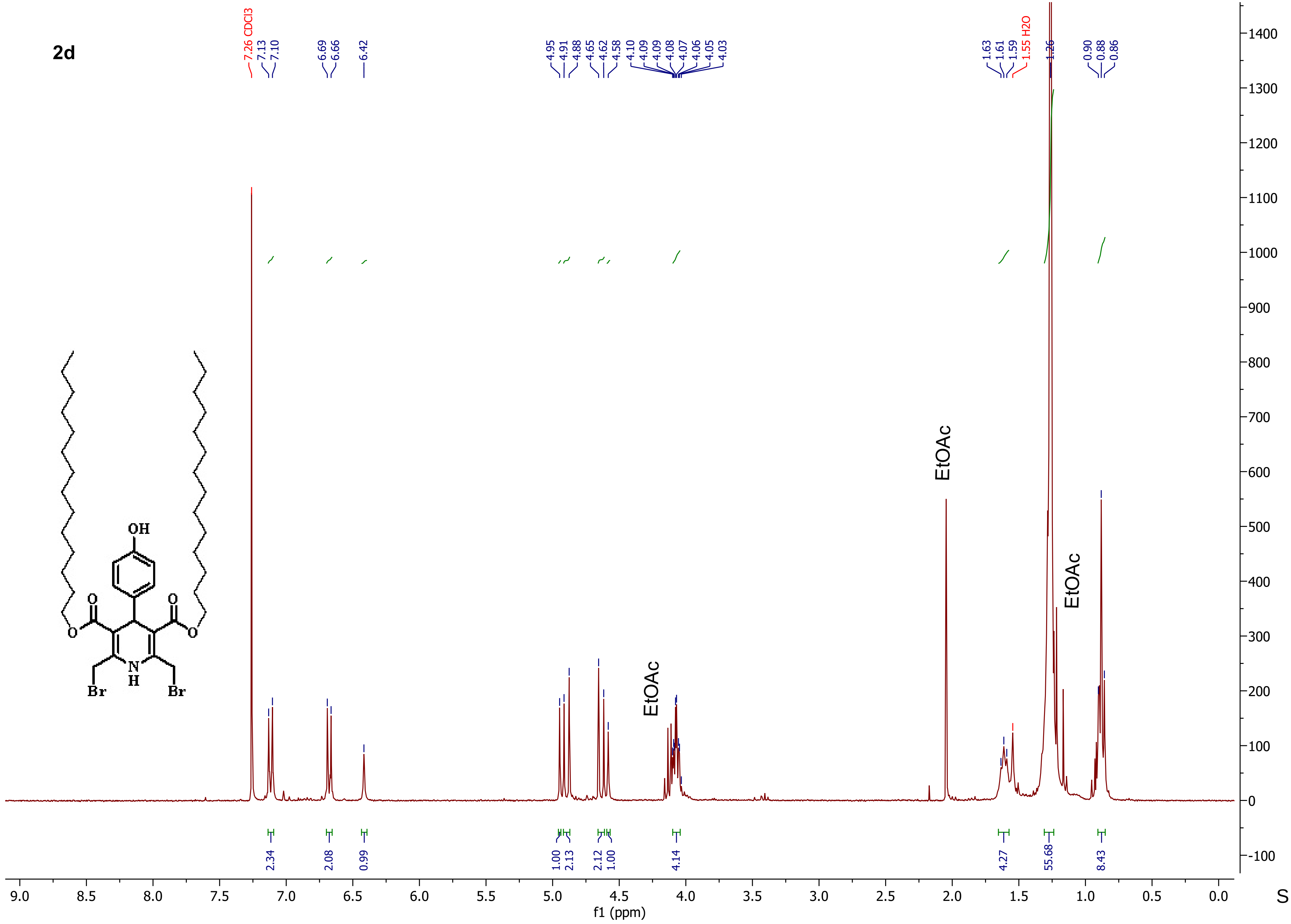
2d
7.26 CDCl3
7.13
7.10
6.69
6.66
6.42
4.95
4.91
4.88
4.65
4.62
4.58
4.10
4.09
4.09
4.08
4.07
4.06
4.05
4.03
1.63
1.61
1.59
1.55 H2O
1.26
0.90
0.88
0.86
OH
O
O
O
O
N
H
Br
Br
EtOAc
EtOAc
EtOAc
2.34
2.08
0.99
1.00
2.13
2.12
1.00
4.14
4.27
55.68
8.43
f1 (ppm)

2d

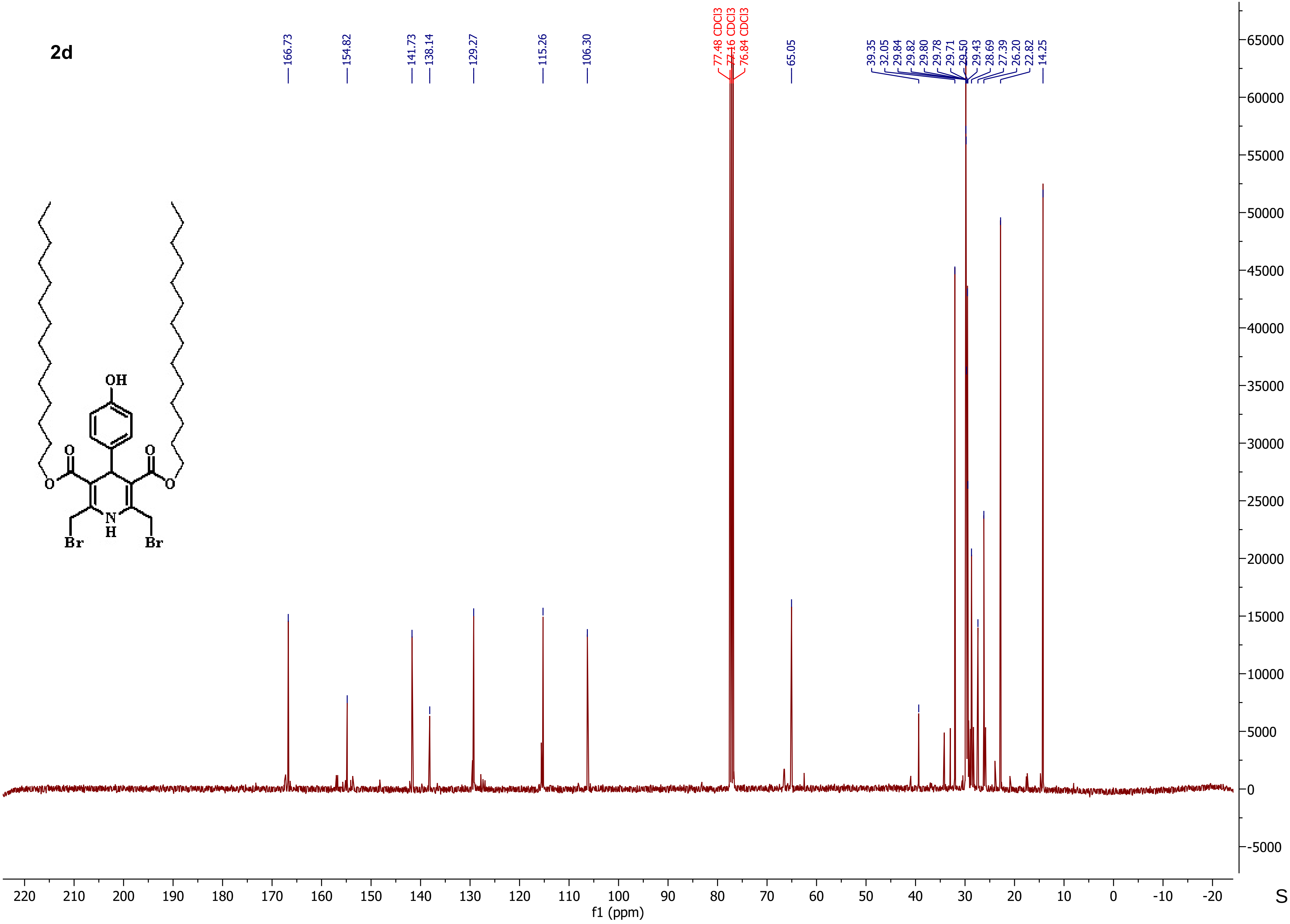

166.73
154.82
141.73
138.14
129.27
115.26
106.30
77.48 CDCl3
77.16 CDCl3
76.84 CDCl3
65.05
39.35
32.05
29.84
29.82
29.80
29.78
29.71
29.50
29.43
28.69
27.39
26.20
22.82
14.25
OH
O
O
N
H
Br
Br
65000
60000
55000
50000
45000
40000
35000
30000
25000
20000
15000
10000
5000
0
-5000
220 210 200 190 180 170 160 150 140 130 120 110 100 90 80 70 60 50 40 30 20 10 0 -10 -20
f1 (ppm)

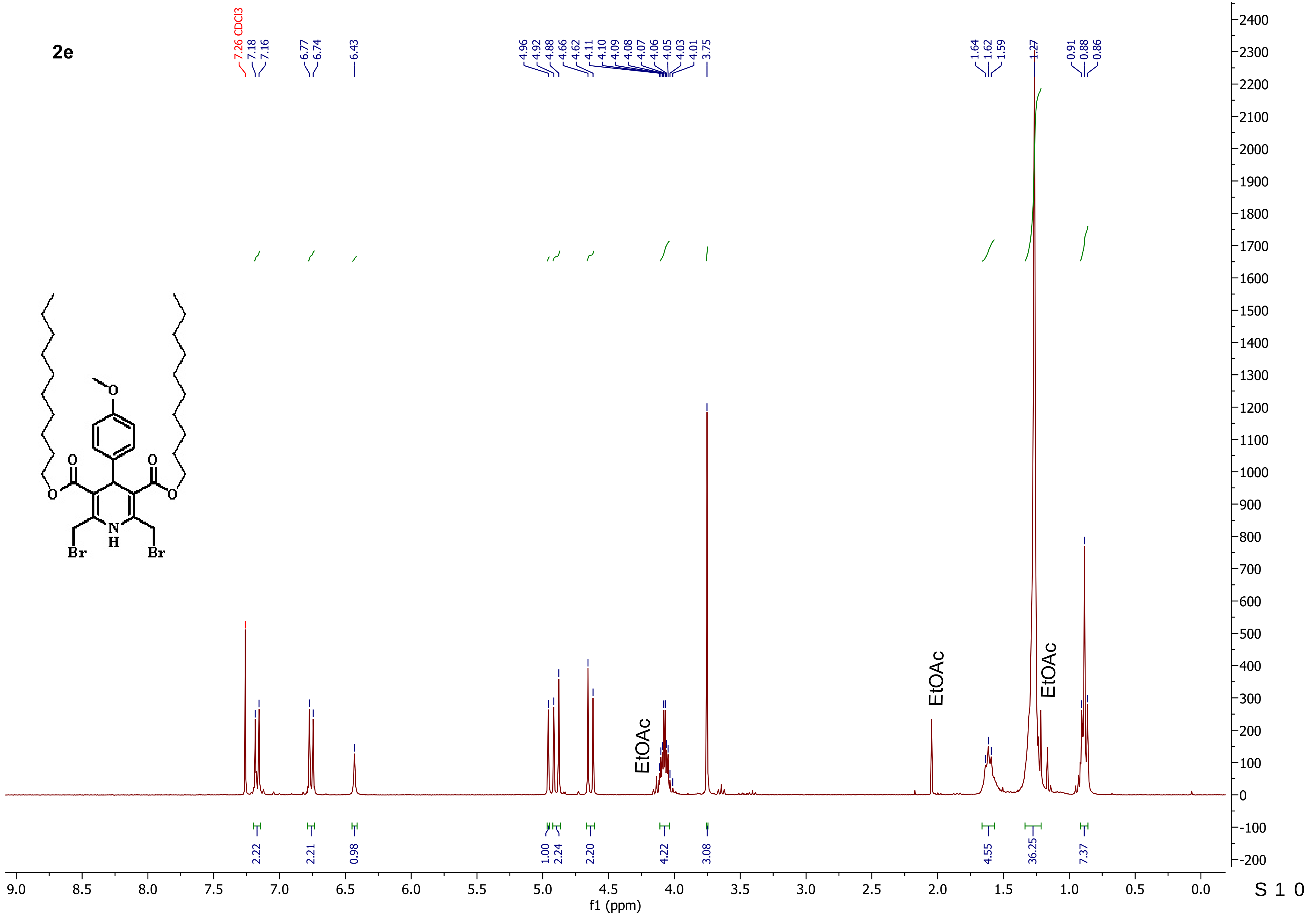

2e
7.26 CDCl3
7.18
7.16
6.77
6.74
6.43
4.96
4.92
4.88
4.66
4.62
4.11
4.10
4.09
4.08
4.07
4.06
4.05
4.03
4.01
3.75
1.64
1.62
1.59
1.27
0.91
0.88
0.86
EtOAc
EtOAc
EtOAc
2.22
2.21
0.98
1.00
2.24
2.20
4.22
3.08
4.55
36.25
7.37
f1 (ppm)

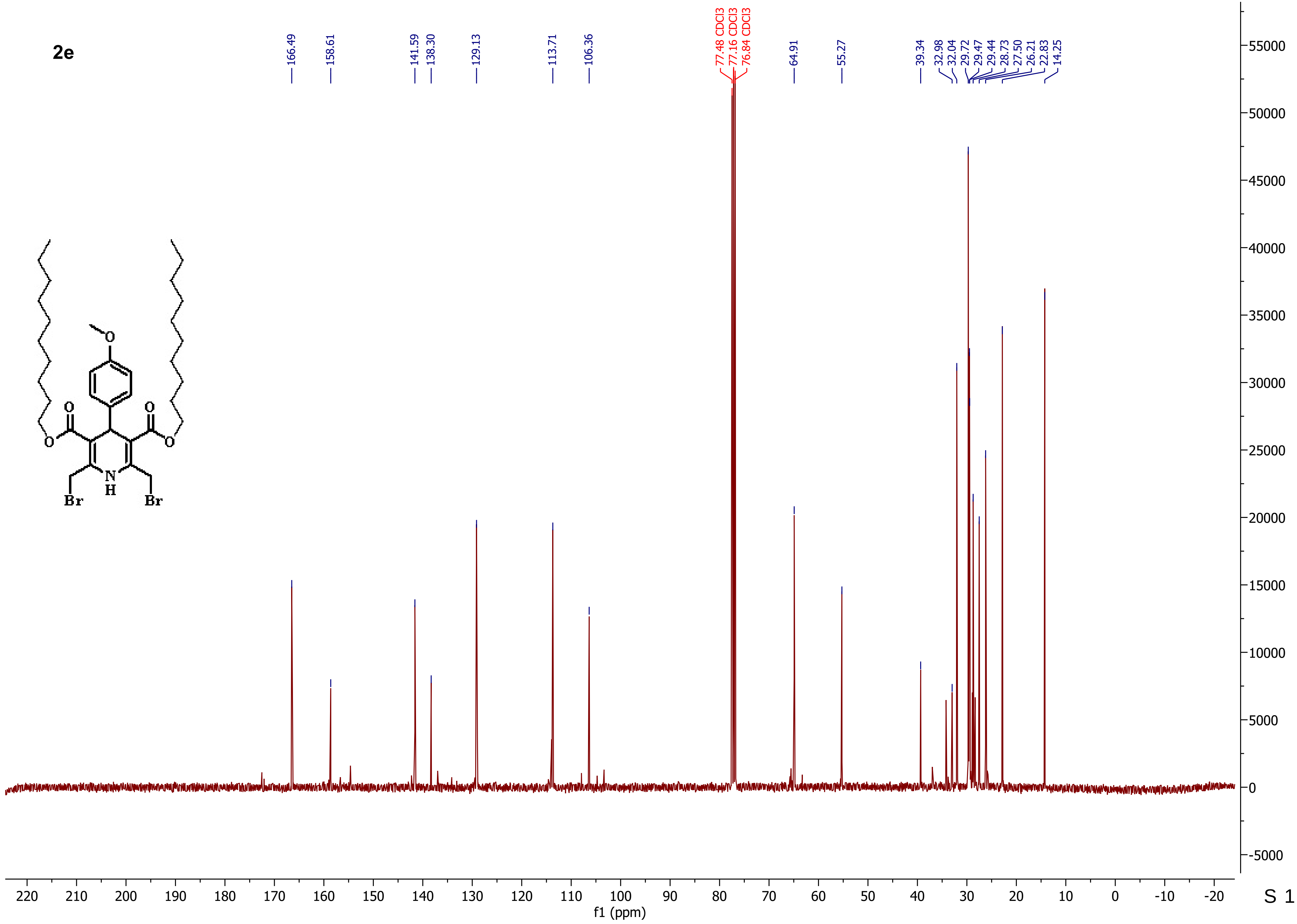
2e
166.49
158.61
141.59
138.30
129.13
113.71
106.36
77.48 CDCl3
77.16 CDCl3
76.84 CDCl3
64.91
55.27
39.34
32.98
32.04
29.72
29.47
29.44
28.73
27.50
26.21
22.83
14.25
f1 (ppm)

2f

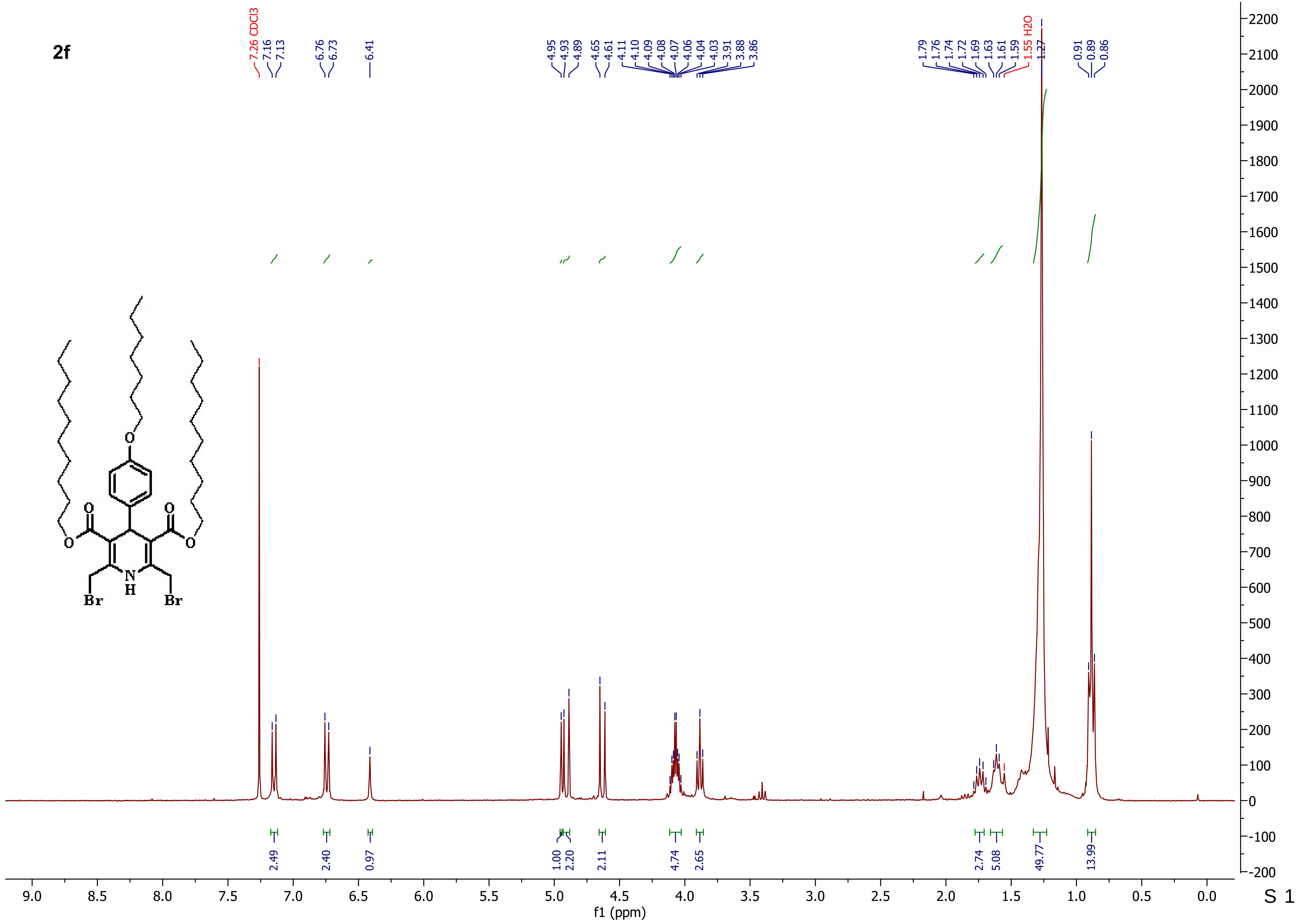
7.26 CDCl3
7.16
7.13
6.76
6.73
6.41
4.95
4.93
4.89
4.65
4.61
4.11
4.10
4.09
4.08
4.07
4.06
4.04
4.03
3.91
3.88
3.86
1.79
1.76
1.74
1.72
1.69
1.63
1.61
1.59
1.55 H2O
1.27
0.91
0.89
0.86
2.49
2.40
0.97
1.00
2.20
2.11
4.74
2.65
2.74
5.08
49.77
13.99
f1 (ppm)
O
O
O
O
O
N
H
Br
Br

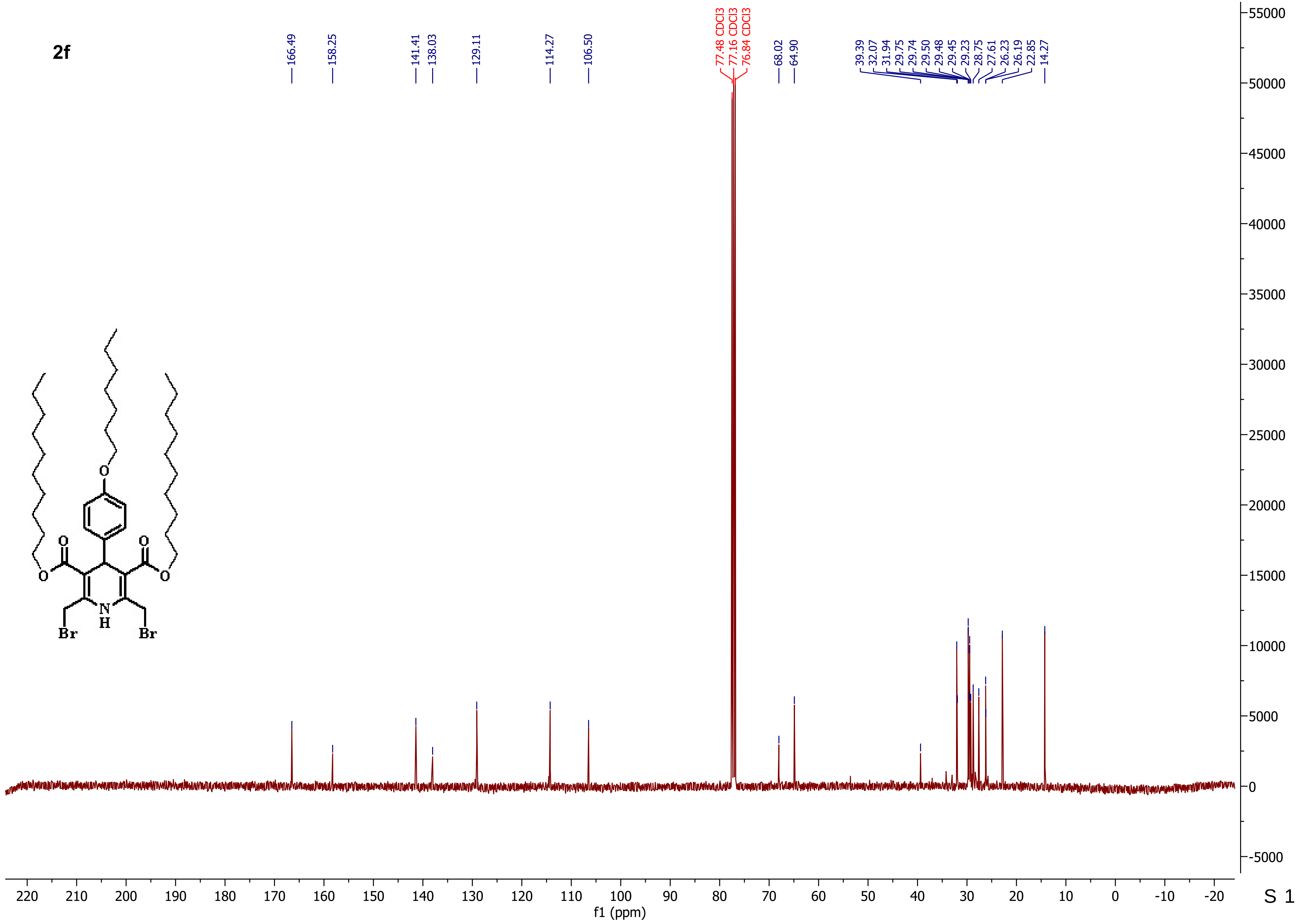
2f
166.49
158.25
141.41
138.03
129.11
114.27
106.50
77.48 CDCl3
77.16 CDCl3
76.84 CDCl3
68.02
64.90
39.39
32.07
31.94
29.75
29.74
29.50
29.48
29.45
29.23
28.75
27.61
26.23
26.19
22.85
14.27
f1 (ppm)
